# Novel scanning procedure enabling the vectorization of entire rhizotron-grown root systems

**DOI:** 10.1186/1746-4811-9-1

**Published:** 2013-01-04

**Authors:** Guillaume Lobet, Xavier Draye

**Affiliations:** 1 Earth and Life Institute, Université catholique de Louvain, Croix du Sud L7.05.11, 1348 Louvain-la-Neuve, Belgium

## Abstract

This paper presents an original spit-and-combine imaging procedure that enables the complete vectorization of complex root systems grown in rhizotrons. The general principle of the method is to (1) separate the root system into a small number of large pieces to reduce root overlap, (2) scan these pieces one by one, (3) analyze separate images with a root tracing software and (4) combine all tracings into a single vectorized root system. This method generates a rich dataset containing morphological, topological and geometrical information of entire root systems grown in rhizotrons. The utility of the method is illustrated with a detailed architectural analysis of a 20-day old maize root system, coupled with a spatial analysis of water uptake patterns.

## Background

Root systems are responsible for the capture of below-ground resources such as nutrients and water. As such, they are thought to be play a central role in the yield establishment of crop plants [1-3]. The availability of a given resource for the plant can be seen as the integration of soil and roots bio-physical constraints. On the one side, the resource distribution in the soil and its mobility defines its *potential* availability for the plant. On the other side, the root system architecture (RSA, including morphology and topology) and the root placement in the soil domain (spatial correlation between the roots and the resource) defines the *actual* resource availability for the plant [4]. However, root and soil constraints do not add independently and emerging behaviors are likely to arise from the non linearity of the soil-root system [5,6]. Therefore, detailed datasets containing root system architecture, root placement and soil resource dynamics are required to improve our understanding of resource capture by plant roots.

Unfortunately, few techniques allow the simultaneous acquisition of precise soil and root information. Precise quantification of the root systems can be done by using, for instance, X-ray computed tomography [7,8], magnetic resonance imaging [9] or transparent artificial soils [10]. Among more classical techniques, growing plants in flat transparent culture boxes (rhizotrons) is widely used in root research. This simple technique provides an easy way to observe the growth and development of a large number of plants in a soil-like substrate [11]. Moreover, rhizotrons allow some level of soil observation, as with the light transmission imaging [12] or neutron radiography [13]. These enable a fine analysis of soil-root relations, given that sufficient information is obtained about the plant and the soil.

**Figure 1:**
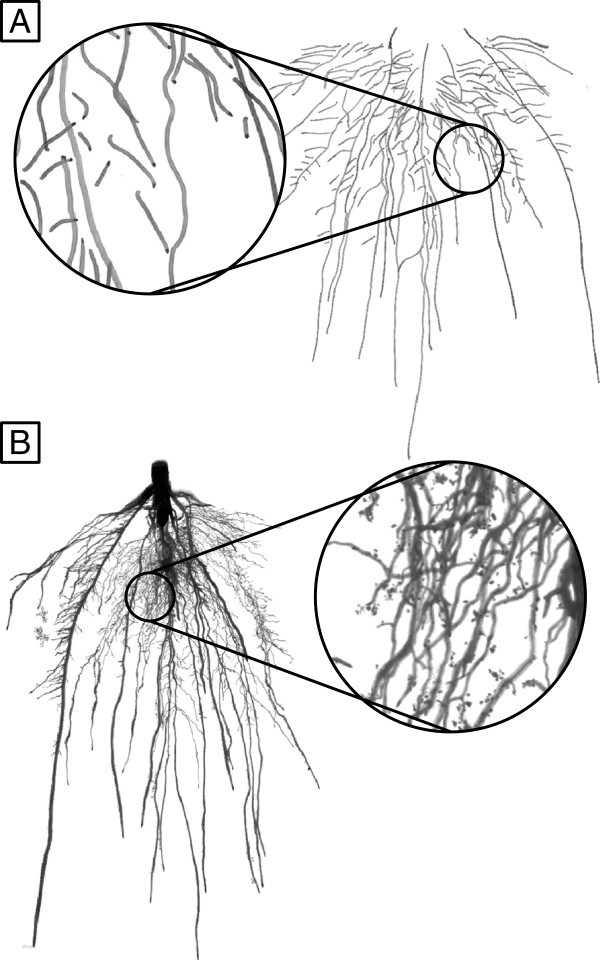
**Comparison of the different root architecture analysis methods.** Comparison of the different root architecture analysis methods for plants grown in rhizotrons. **A.** Hand drawing of a 20-days old maize root system inside a rhizotron. The closeup shows disconnected root segments on the drawing. **B.** Same root system scanned after the experiment. The closeup shows roots overlapping and crossing.

The description of the root system of plants grown in rhizotron is often performed by recording the visible roots on the outer surface of the rhizotron (by scanning or taking pictures of the rhizotron surface or manually drawing the visible roots, *in-situ* images, Figure 1A), or, more rarely, by removing the substrate from the rhizotron and scanning the root system (*ex-situ* images, Figure 1B). These methods yield complementary information about the root system architecture (Table 2), but neither of them provide precise data about the root morphology, topology and placement that is required for a local analysis of soil-root interactions. The analysis of rhizotrons images is often restricted to a limited number of variables, often specific to the image analysis software (for an exhaustive presentation of the existing root image analysis software, see http://www.root-image-analysis.org).

A general restriction of the currently available root image analysis softwares lies in the difficulty to analyze highly branched root systems with a large degree of root overlap in 2D (Figure 1B, close-up). Not surprisingly, a trade-off is usually observed between the degree of automation and the level of detail obtained. Among existing software, SmartRoot [14] provides, to our knowledge, the most exhaustive root architecture dataset, containing detailed morphological, topological, spatial and temporal information. In addition, the SmartRoot dataset enables more complex architectural analysis [15]. For example, Fitter’s architectural indexes can be computed from topological information [16] or dynamic traits such as the growth rates of first and second order roots can be inferred from static topological and morphological traits [17].

We propose here a new hybrid method that combines the strengths of existing techniques and software in order to generate detailed and spatially correct vector-based versions of entire root systems (Table 1).

**Table 1:** Comparison of the different methods

**Method**	**all roots**	**topology**	**diameter**	**length**	**position**	**time-series**	**overlap**
*In-situ*	no	some	yes/no	some	**yes**	**yes**	some
*Ex-situ*	**yes**	**yes**	**yes**	**yes**	no	no	yes
Hybrid	**yes**	**yes**	**yes**	**yes**	**yes**	**yes**	**no**

Comparison of the different root architecture analysis methods of plants grown in rhizotrons.

**Figure 2:**
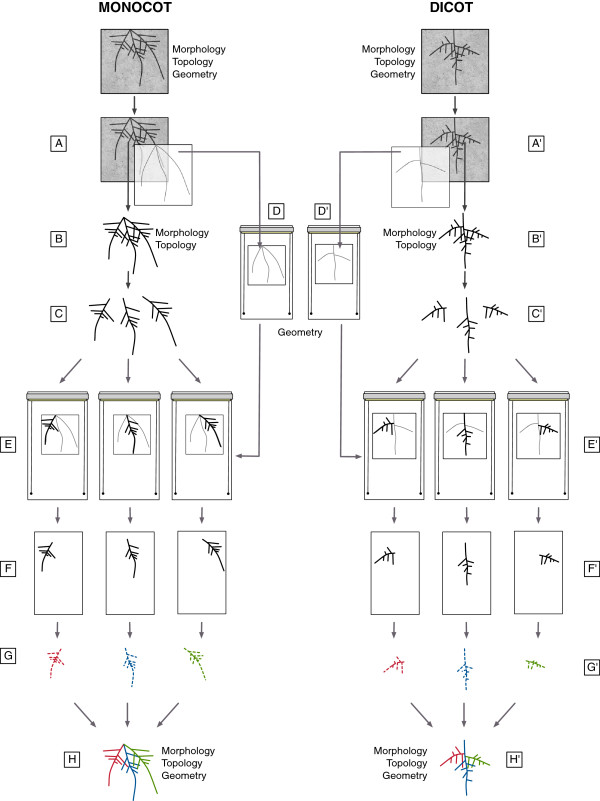
**Procedure to digitize an entire root system.** Procedure to vectorize an entire root system. **A.** Thick and easy to recognize parts of the root system are manually traced on a transparent sheet at the rhizotron surface. **B.** The entire root system is extracted and cleaned. **C.** The different parts are dissociated. **D.** The transparent sheet is laid on the surface of the scanner. **E.** Each root axis is successively placed on the scanner according to the drawing and scanned. **F.** As a result, every root system is contained in a set of several images. **G.** Every root image is traced with SmartRoot. **H.** The different tracing are regroup into a single vectorized root system using SmartRoot.

In practice, major root axes appearing at the rhizotron transparent side are manually traced on an acetate sheet superimposed on the rhizotron (Figure 2A). The rhizotron is then opened and the root system is extracted and split into parts where root overlap is minimized (Figure 2B and C). In monocots, one would typically split the root system in first order roots (primary, seminal, adventitious) while in dicots, one would separate the tap root from its large branches.

The acetate sheet (obtained *in situ*) is then attached on the glass of the scanner (Figure 2D) and, one by one, each root part is laid on the scanner, aligned to its corresponding tracing and scanned (Figure 2E). The whole scanning procedure yields a set of high resolution registered images where roots (1) are positioned as in the rhizotron and (2) display a much reduced degree of root overlap. The subsequent tracing of the roots is realized with SmartRoot (Figure 2F) on the individual images and the resulting morphological datasets are combined into a unique and complete vectorized root system.

## Results and discussions

The ability of the method to generate vectorized versions of entire root systems and its utility in the framework of soil-root-interaction research is demonstrated with the analysis of water uptake dynamics in maize. Plants were grown in thin rhizotrons under non-limiting conditions until emergence of the sixth leave. The nutrient solution supply was then interrupted and the evolution of the 2D soil water content was monitored during three days using light transmission imaging [12].

### Step 1: Architectural analysis of complex root systems

**Figure 3:**
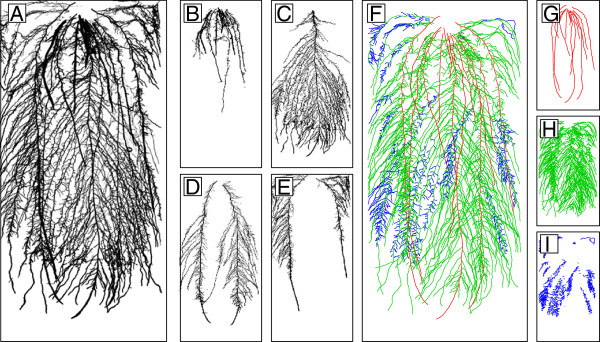
**Example of vectorized maize root system.** Example of vectorized maize root system. **A.** Complete scan. **B-E.** Individual root scans. **F.** Entire vectorized root system. **G-I.** Vectorized root system by root order. **F-I.** Different colors represent different root orders: red = 1, green = 2, blue = 3.

**Figure 4:**
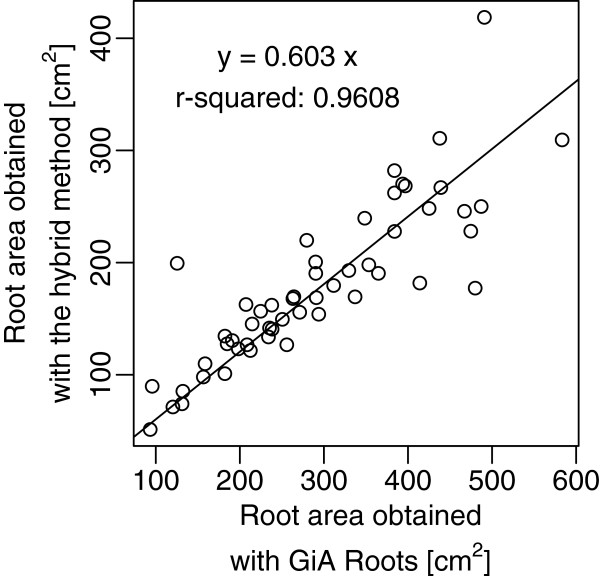
**Scanned vs traced root system area.** Comparison of the total root surface obtained with a classical root scan analysis (performed with GiARoots [18]) and the hybrid method.

**Figure 5:**
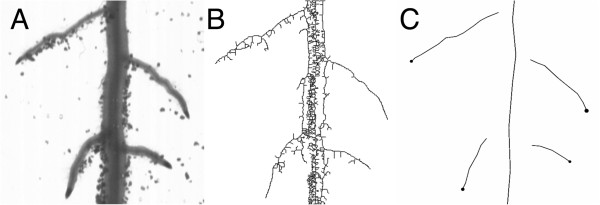
**Skeleton comparison between GiA Roots and SmartRoot.** Skeleton comparison between GiA Roots and SmartRoot. **A.** Original image. **B.** Skeleton obtained with GiA Root. **C.** Skeleton obtained with SmartRoot.

The hybrid method was successfully used to create vectorized versions of entire root systems of 20-day old maize plants having 2-3 seminal roots and 2-4 adventitious roots (Figure 3A-E). To validate the accuracy of the method, the total root area estimated for 52 plants were compared with the area obtained from classical *ex-situ* images (similar to the one presented in the Figure 3A) using GiA Roots [18] (Figure 4). The high determination coefficient (linear regression without intercept, r^2^=0.96) between the two methods indicates that the results obtained with the hybrid method are within the range of classically obtained data. It is interesting to see that values obtained with GiA Roots tend to be overestimated compared to the hybrid method. This is probably due to two main factors. Firstly, with the semi-automated SmartRoot, precise tracings are obtained, but some roots may have been missed. Secondly, the reduced quality of the original root images have generated several false positive during the GiA Roots thresholding/skeletonizing steps. Both factors would contribute to the total root length difference between the two methods (Figure 5).

**Figure 6:**
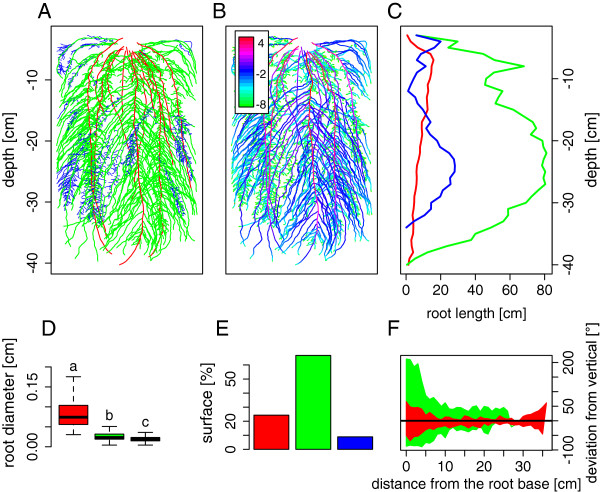
**Root system architecture results.** Root system architecture results. **A.** Entire vectorized root system. **B.** Total root surface distal any position in the root system (scale is in log(cm^2^)). **C.** Root length profiles for the different root orders. **D.** Comparison of the root diameters for the different root orders (values suffixed with the same letter are not significantly different at p<0.01, t-test). **E.** Surface proportion of the different root orders. **F.** Orientation of the roots along their root axis. The horizontal black line highlight the vertical direction. In all the figures (except B), different colors represent different root order: red = 1, green = 2, blue = 3.

The dataset resulting from the hybrid method was analyzed using common statistical software (data presented here were analyzed using R [19]). Figure 6 highlights some of the information that can be extracted from the SmartRoot database: a complete representation of the root system (by root orders, Figure 6A), a representation of the total root surface distal any position in the root system (Figure 6B), the comparison of the root length 1D profiles of the different root orders (Figure 6C), the comparison of the root diameters for the different root orders (Figure 6D), the contribution of the different root orders to the global root surface (Figure 6E) and the changes in orientation of the roots along their axis.

These different variables are highly informative in the framework of root water uptake research. For instance, assuming that the cumulative volume (Figure 6B) is proportional to the quantity of water crossing the segments, Figure 6B highlights the large differences in terms of axial water flow between the first and second order roots (up the 12 orders of magnitudes). This assumption is supported by the fact that first order roots have much thicker roots (Figure 6D) with larger cross-sectional xylem area. On the other hand, the second and third order roots have much thinner root (Figure 6D), but represent the majority of the total root length and surface (Figure 6C and E). Moreover, these roots tend to have a lesser gravitropic behavior (Figure 6F), ensuring a better exploration of the horizontal soil layers. The root system can therefore be divided in two functional type of roots: the primary roots, responsible for (1) the vertical exploration of the soil and (2) the majority of the axial transport of water to the shoot, and the lesser order roots responsible for (1) the horizontal exploration of the soil layers and (2) the majority of the water extraction.

### Step 2: Local analysis of root-soil interactions

As mentioned earlier, a detailed description of root system architecture (including root placement) and quantification of the distribution of the observed resources in the soil domain is required to analyze the interplay between root systems and their environment [4-6]. Because the vectorized root system is spatially registered on the rhizotron surface, the architectural information can be combined with any 2D description of soil resources. Here, we crossed the SmartRoot dataset with 2D maps of soil water content obtained with the light transmission imaging technique [12].

**Figure 7:**
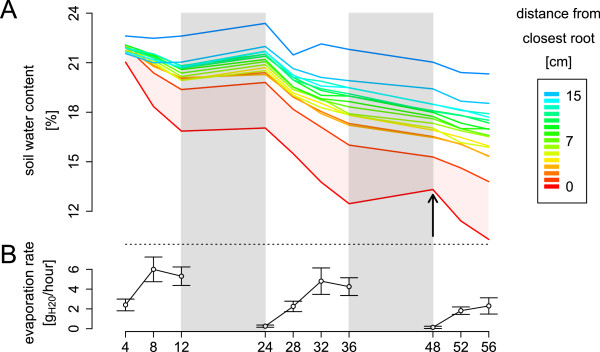
**Local analysis of soil root interactions.A.** Evolution of transpiration rate during the three days of experiments. **B.** Evolution of the soil water content at different distances from the roots (0-15 cm). Red area highlight the difference in soil water content between the root and the first centimeter of soil. Grey area represent the night (no measurements). The arrow highlight the water redistribution in the soil occurring at night.

Figure 7A shows the temporal evolution of the soil water content as a function of the distance to the closest root. This estimation combines the root placement information and the local evolution of soil water content around individual roots. The figure highlighted the strong water content gradient appearing at the vicinity of the roots (Figure 7A, red area). This non-linear decrease of water content at the proximity of the roots results in a higher resistance to water flow compared to the bulk soil and can ultimately lead to local hydraulic isolation of the root system [6,20] and reduction of transpiration (Figure 7A). Additionally, the same figure shows the apparition of a refilling process occurring during the second night between the bulk soil and the dry region close to the roots (Figure 7B, arrow). This example highlights the interest of the method in the framework of soil-root interaction analysis at a local scale (in this example, at the centimeter scale).

Depending on the size and complexity of the root system, this method can be time consuming and is not amenable even to moderate throughput. In our example, the image acquisition step (cleaning and scanning) took between 20 to 50 minutes per root system. The time required for root tracing with SmartRoot ranged from 1 to 4 hours. Nevertheless, the methodology proves to be a valuable tools to analyze complex root systems since, to the authors knowledge, no other methods today is able to extract such detailed dataset from entire root systems well beyond the seedling stage.

## Conclusion

We have presented a new method based on a multiple scan approach to vectorize entire root systems grown in rhizotrons. This methods combines the strengths of two classical methods: *in situ* recording of root placement and *ex situ* high resolution scanning of root system fragments. The method yields ultimately a rich dataset containing detailed information on every root (position, morphology and topology) which can be easily crossed with spatial soil information data to analyze the interplay between the root system and local soil conditions. The method has been successfully used to vectorize root systems of 20-day old maize plants and has been used for the analysis of spatial root water uptake patterns.

Despite the time required by the method (both root scan and image analysis), we believe that it opens new perspectives for root-soil research. It proved an affordable way to precisely describe complex root system architecture and their interplay with their direct environment. We are currently using it for the parametrization of functional-structural plant models that simulate water and nutrient movement in the soil-root domain [21-25]. Using the digital structure created with the hybrid method, these models could be used to analyze *in silico* the water dynamics of the system.

The hybrid approach is neither bound to a specific acquisition device nor to a specific image analysis software. It provides a framework which should improve with future technical advances (e.g. faster scanners) and software developments (e.g. increased tracing automation). Following the root tracing protocol described by [26] the method could also be extended to the temporal analysis of root growth and its merging with relevant local soil conditions (e.g. soil water content and mechanical impedance).

Finally, the method presented here could be extended to generate precise estimation of topological and morphological traits of field-grown root systems, although some aspect of the root placement information would be lost.

## Methods

### Hybrid method

#### Plant culture

Twenty maize plants (B73) were grown during 20 days in thin rhizotrons (50 x 50 x 0.4 cm [27]) made of transparent acrylic filled with a mix of white sand (98.5%) and hectorite clay (Bentone SA, 1.4%). The substrate was maintained at field capacity with a modified Hoagland solution (doubled iron content, Table 2). In order to increase the number of roots growing along the rhizotron surface, rhizotrons were stored at an angle of ≈ 35° [28,29].

**Table 2:** Modified Hoagland solution

**Component**		**Stock Solution**	**mL/1L**
2M KNO_3_		202g/L	2.5
2M Ca(NO_3_)_2_ × 4H_2_O		236g/0.5L	2.5
FeEDTA		15g/L	1.5 **× 2** = 3
2M MgSO_4_ × 7H_2_O		493g/L	1
1M NH_4_NO_3_		80g/L	1
Minors:			1
	H_3_BO_3_	2.86g/L	
	MnCl_2_ × 4H2O	1.81g/L	
	ZnSO_4_ × 7H2O	0.22g/L	
	CuSO_4_	0.051g/L	
	H3MoO_4_ × H_2_O **or**	0.09g/L	
	Na_2_MoO_4_ × 2H_2_O	0.12g/L	
1M KH_2_PO_4_		136g/L	0.5
(pH 6.0 w/ 3M KOH)			
	KOH 3M	168,3g/L	

Composition of the modified Hoagland solution. Iron content (FeEDTA) has been doubled.

#### *In situ* tracing of the root axis

At the end of the growing period, before removal of the plant from the rhizotrons, the visible roots were manually traced on a transparent sheet (e.g. Avery 2503 Transparents JE 90 Microns, but any transparent sheet works) placed on the rhizotron surface (Figure 2A). Different roots orders were drawn using different colors (Stabilo OHPen universal, red and green) for an easier placement of the roots on the scanner (see below). We used light colors that do not appear on the final scan.

#### Root system preparation

After the tracing, rhizotrons were open and plants were taken out. Root systems were separated from the shoot and cleaned from the substrate (Figure 2B). Root cleaning was performed by soaking them during 5 minutes into water with a mild detergent. This procedure has the advantage of removing the majority of the soil particles without breaking the different roots. Finer soil particles still attached to the root were removed using a small painting brush. Plant were stored in a 50% ethanol solution before the scanning procedure.

#### Individual root scan

**Figure 8:**
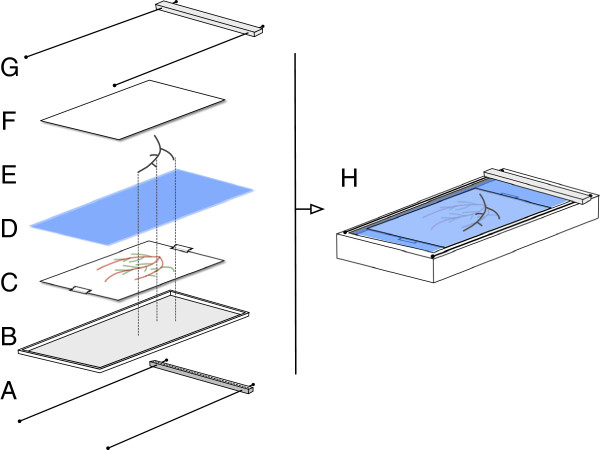
**Scanning procedure.** Scanning procedure. **A.** Flat-bed scanner. **B.** Transparent sheet with the root tracing. **C.** Water. **D.** Part of root system. **E.** Transparent sheet for reduction of shading effect. **F.** Complete setup. **G.** Scanner details. Parts of root systems are replaced according to the root tracing (dotted lines).

The scanning procedure was performed with a custom flat bed scanner whose scanning window can be filled with water to enable an easier positioning of the roots (Figure 8A and C). The scanner (Medion 3600 DPI) was customized in house in such way that the light source and the sensor on both side of a large water container (21 x 60 cm, Figure 8G). The transparent film with the root tracing was first fixed at the bottom of the scanner with transparent adhesive paper (Figure 8B). Then, the root system was split into fragments that were scanned individually. Each fragment (usually corresponding to a first order roots) was placed on the scanning window according to its position on the root tracing (Figure 2C-E and Figure 8D). Since every one of these roots is different (in length and shape), the correct position of every axis is found easily. Once the fragment was positioned, its lateral roots were carefully untangled (with a painting brush if needed). The use of a submersible scanning window streamlined the process as the roots tended to recover their initial shape in the water. A transparent plastic sheet (identical to the one used for the root drawing) was placed on the water surface to improve image quality and reduce shadows around the roots (Figure 8E). The scan was done with a 600 DPI resolution. The same procedure was repeated for every fragment of the root system. The final output of the scanning was therefore an image set of *x* images, where *x* is the number of fragments of the root system.

#### Root vectorization

Every root image was analyzed using the root image analysis software SmartRoot [14] (Figure 2F). The software enables a semi-automated tracing of individual roots and generates morphological and topological information. In order to streamline the tracing of complete root systems, several new tools were implemented in SmartRoot which allow the user to perform actions simultaneously on multiple roots: Root list panel A new tool was implemented that displays the different roots as a hierarchical list (every root being nested in its parent node). This tool allows a fast observation of basic root statistics and an easy selection of multiple roots (even not adjacent in the image). Action on multiple roots Complementary to the multiple root selection tool in the root list panel, the possibility to perform actions on multiple roots was implemented. These actions, that were applied on single roots in previous versions, include the deletion of roots, their attachment to a common parent or the detection of lateral roots. Import multiple files A new import function was implemented to allow the import of multiple datafiles (e.g. those from the different root scans) into a single image. This tool enables the reconstruction of a complete root system.

Using these tools, the root tracing with SmartRoot is performed using the following procedure: (1) trace the roots on the different images, (2) import the different tracings into a single image, (3) connect the roots from the different images and (4) export the tracing to a single database. A screencast detailing the different steps in the root tracing procedure is available at the address: http://www.uclouvain.be/smartroot

### Local analysis of root-soil interactions

**Figure 9:**
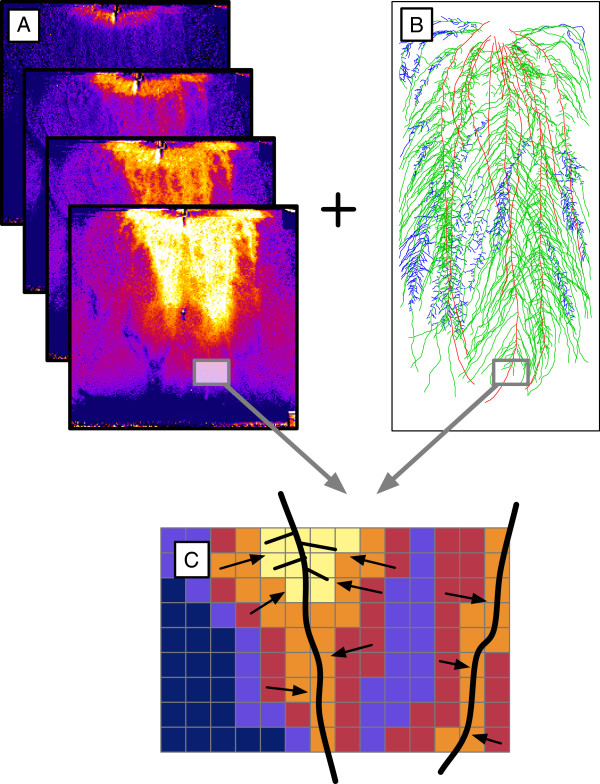
**Local uptake analysis.** Local uptake analysis. **A.** Time-series images of soil water content distribution. **B.** Vectorized root system. **C.** Local analysis of the variation in the soil water content using the information obtained with the vectorized root system. Pixels are oversized for illustration purpose.

Time series of 2D maps of soil water content (Figure 9A) were obtained using the light transmission imaging technique [12]. Briefly, the technique relies on the relationship between the water content of the substrate and its light transmission properties [30]. Using this principle, the 2D soil water content distribution was obtained by placing every rhizotron between a light source (light tubes, 36W, Sylvania Standard F36W/33-640-T8) and a regular CCD camera (Canon EOS 450D with a lens Canon EF 50mm 1:1.8). The time series consisted in a light transmission image every four hours during three days.

The obtained time-series were analyzed by crossing the data contained in the light transmission images with the vectorized root system (Figure 9B and C). This was achieved by (1) reducing the image to 50 x 50 pixels so that every pixels had a size of 1 cm^2^, (2) finding, for every pixel, the closest root segment (euclidian distance) and (3) creating a new database merging the soil water content and root information. Root growth was assumed to be negligible during the three days of measurement.

## Authors’ contributions

XD and GL conceived the study. XD participated in its design and coordination. GL performed the different experiments and the data analysis. XD and GL wrote the manuscript. All authors read and approved the final manuscript.
